# Clinical Outcome of Third-Line Pazopanib in a Patient with Metastatic Renal Cell Carcinoma

**DOI:** 10.1155/2015/629046

**Published:** 2015-12-20

**Authors:** Michela Roberto, Maria Bassanelli, Elsa Iannicelli, Silvana Giacinti, Chiara D'Antonio, Anna Maria Aschelter, Paolo Marchetti

**Affiliations:** ^1^Department of Clinical and Molecular Medicine, Faculty of Medicine and Psychology “Sapienza”, St. Andrea Hospital, Via di Grottarossa 1035-1030, 00189 Rome, Italy; ^2^Department of Radiology, Faculty of Medicine and Psychology “Sapienza”, St. Andrea Hospital, Via di Grottarossa 1035-1030, 00189 Rome, Italy; ^3^Department of Medical and Surgical Sciences and Translational Medicine, Faculty of Medicine and Psychology “Sapienza”, St. Andrea Hospital, Via di Grottarossa 1035-1030, 00189 Rome, Italy

## Abstract

*Background.* Renal cell carcinoma accounts for about 2-3% of all malignant tumors. The prevalence of brain metastases from RCC is less than 20% of cases. Traditionally, whole brain radiotherapy as well as the latest stereotactic radiosurgery improves both survival and local tumor control. These treatments also allow stabilization of clinical symptomatology. However, validated treatment guidelines for RCC patients with brain metastases are not yet available on account of the frequent exclusion of such patients from clinical trials. Moreover, limited data about the sequential use of three therapies, changing the class of agent, have been published up to now.* Case Report.* We report the case of a patient with metastatic RCC who developed disease progression after sunitinib and everolimus as first-line and second-line therapy, respectively. Thus, he underwent a multimodality treatment with pazopanib, as third-line therapy, to control systemic disease and radiosurgery directed on the new brain metastasis. To date, the patient is still receiving pazopanib, with progression-free survival and overall survival of 43 and 103 months, respectively.* Conclusion.* In a context characterized by different emerging options, with no general consensus on the optimal treatment strategy, the use of pazopanib in pretreated patients could be a suitable choice.

## 1. Introduction

Renal cell carcinoma (RCC) accounts for about 2-3% of all adult, malignant tumors. Metastatic disease frequently occurs, about 50% of the cases, and a large analysis shows that the most common sites of metastases are lung, bone, lymph nodes, and liver (50, 40, 25, and 20% of cases, resp.). Adrenal and brain metastases are rarely diagnosed (about 8–10% of the patients) [1].

According to the Memorial Sloan-Kettering Cancer Center (MSKCC), three prognostic risk groups can be distinguished: favorable, intermediate, and poor [2]. Patients with advanced RCC belonging to the favorable risk group have median overall survival (OS) of 43 months; those belonging to the intermediate and poor groups have 27 and 8.8 months, respectively.

However, in the presence of brain metastases (BM), the prognosis of RCC patients worsens, with a median OS not reaching 20 months using traditional whole-brain radiotherapy (WBRT) alone [3].

Here we describe a case of long-surviving patient who experienced progression of disease after two previous lines of treatment. He underwent a multimodality treatment, consisting of stereotactic radiosurgery (SRS) of the BM and pazopanib as third-line therapy, with a good clinical outcome.

## 2. Case Report

**Figure 1:**
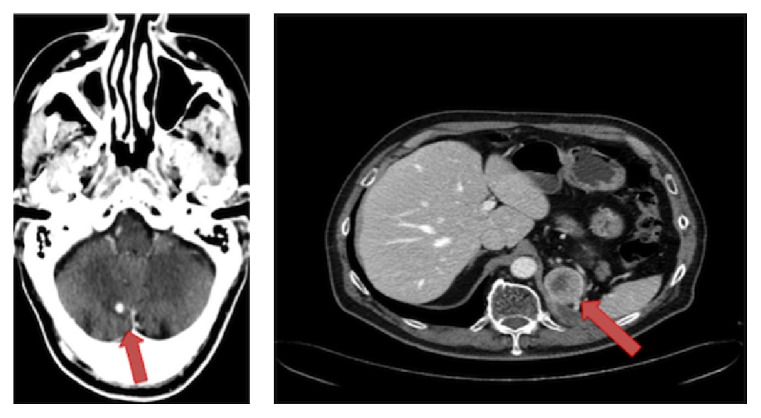
Before starting pazopanib a cerebellar lesion of 6 mm in diameter appeared and left adrenal gland solid nodule was 4 cm in size, with a little necrotic core.

**Figure 2:**
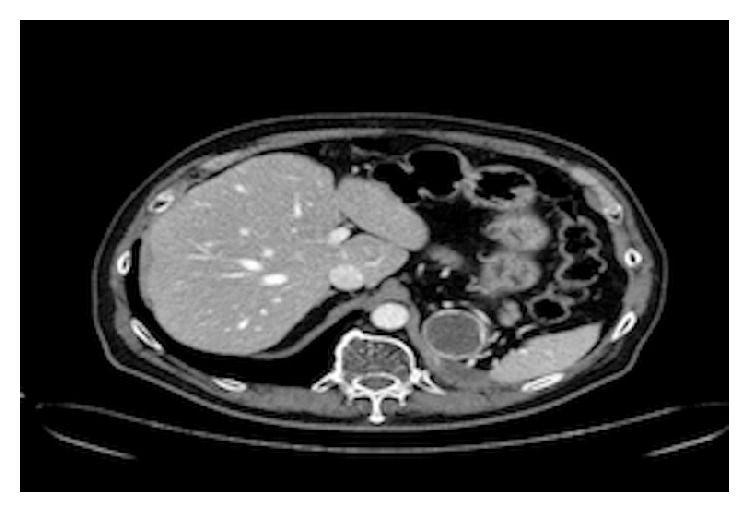
After 3 cycles of pazopanib the adrenal metastasis was stable in dimension but with more necrotic area.

**Figure 3:**
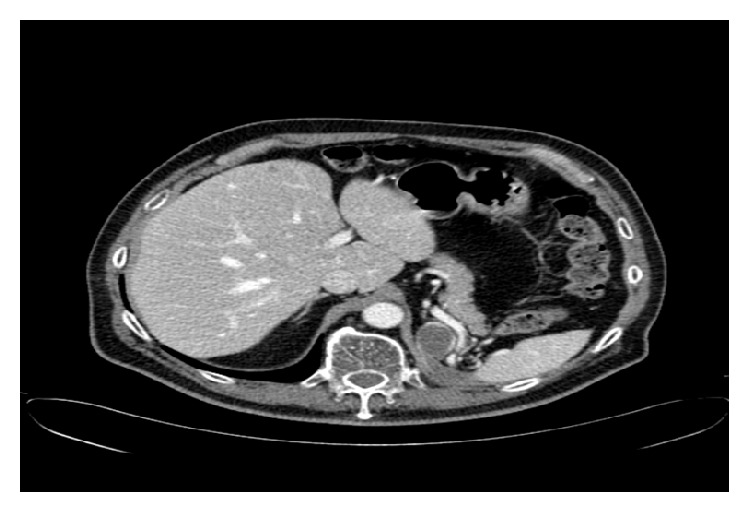
After 12 cycles of pazopanib there was a 30% reduction.

We report the case of a 76 year-old man who underwent a radical left nephrectomy for clear cell RCC (Fuhrman grade 1, stage II according to AJCC) in February 2007. Follow-up was negative until February 2009 when a whole-body computed tomography (CT) scan revealed lung nodule with a major diameter of 2.8 cm in the inferior left lobe. Considering the long disease-free survival time (24 months) along with the presence of single metastasis, a left lung wedge resection was performed. The histology confirmed the diagnosis of metastasis from clear cell RCC. About 5 months later, a spiral CT showed a new malignant micronodule in the right superior lung lobe and nodules ranging from 2.8 to 1.8 cm in the left and right adrenal glands, respectively. Patient's Karnofsky Performance Scale score was 90% and he was classified in the favorable risk group according to both MSKCC and Heng's score criteria. In August 2009 he started a tyrosine-kinase inhibitor (TKI), sunitinib 50 mg per day (4 weeks on and 2 weeks off). After 2 cycles of treatment, a grade 3 mucositis occurred but after 3 weeks of break it declined to grade 1. Thus, he resumed therapy at the lower dosage of 37.5 mg per day (4 weeks on and 2 weeks off). The whole-body CT scan, regularly performed every 3 months, demonstrated a stable disease (SD) as best response until June 2011 when, after 15 cycles of therapy, it revealed a progressive disease (PD) at the left adrenal gland which was confirmed by magnetic resonance imaging. Therefore, in August 2011, a second-line therapy with everolimus 10 mg per day was started. The treatment was stopped after just 4 months because of the fact that CT scan had showed a new cerebellar lesion of 6 mm in the right side and a further progression of the left adrenal nodule (4 cm) (Figure 1). The remaining malignant areas were instead stable. Patient reported grade 2 of asthenia, muscle pain, and edema of the legs during the therapy. However, on account of his good performance status and the long-lasting disease control with sunitinib, we further decided to refer him to a multimodality treatment. Thus, he received another TKI after he had undergone SRS (18 Gy, 1 fr) of the cerebellar lesion. The tolerance to SRS was good and the third-line therapy with the TKI, pazopanib (800 mg per day), was started in February 2012. The first CT evaluation showed SD in both cerebellum and adrenal glands, although the left adrenal nodule had become necrotic (Figure 2). After twelve cycles, 30% reduction of the left adrenal metastasis was observed (Figure 3) with further reduction of 10% in July 2013. The other malignant lesions appeared to be stable. Eighteen cycles were administered at full dosage. Later, dosage was firstly reduced to 600 and then to 400 mg per day for the occurrence of grade 3 hypertension and mucositis. Such a therapy is still in progress with a progression-free survival (PFS) and OS of 43 and 103 months, respectively.

## 3. Discussion

Brain metastases occur in less than 20% of patients with RCC and are generally related to a very poor prognosis [3]. Shuch et al. reported that the median OS after the diagnosis of BM was 10.7 months with a 1-year, 2-year, and 5-year OS rates of 48%, 30%, and 12%, respectively [4].

The WBRT and the latest SRS are demonstrated to improve survival, local tumor control, and clinical symptomatology [5]. However, there are no validated treatment guidelines for patients with BM from RCC on account of the frequent exclusion of such patients from clinical trials [6]. Indeed, results from either expanded access or small retrospective studies are merely available [3, 7, 8]. Nevertheless, these data have showed a trend towards improved survival, mostly achieved by the application of multimodality treatment, which consists of surgery, WBRT, or SRS administration with concomitant or sequential TKI therapy.

We report a case of patient who developed BM after sunitinib as first-line therapy and everolimus as second-line therapy, with PFS of 24 and 4 months, respectively. We referred him to multimodality treatment with SRS of the cerebellar metastasis and pazopanib as third-line therapy. In our opinion, his excellent OS of more than 8 years raises particular interest. Indeed, to the best of our knowledge, none of the few previous studies about second- and third-line metastatic RCC (mRCC) reported similar outcome.

Although there are limited published data on the sequential use of three therapies in the treatment of mRCC, several studies showed an increased disease control as well as improvements in quality of life by changing the class of agent. First of all, the RECORD-1 trial showed a better outcome with everolimus (mTOR inhibitor) versus placebo (PFS was 4 and 1.9 months, resp.) in patients progressing on VEGF inhibitors (VEGFi) [9]. Another phase III study instead showed clinical benefit using sorafenib as third-line therapy [10]. In detail, a significant risk reduction of death (HR = 0.72) and progression (HR = 0.44) in the sorafenib group was reported. Moreover, data from 879 patients treated with several classes of agents as third-line therapies, such as VEGFi and mTOR inhibitors (mTORi), have been reported at recent genitourinary cancers symposium [11]. Overall, the median PFS was 5.1 months and the median OS from beginning third-line therapy was 12.0 months. Similarly, in the GOLD trial, patients previously treated with VEGFi-mTORi sequence and randomized to receive dovitinib or sorafenib as third-line therapy reached a median PFS of 3.7 versus 3.6 months, respectively [12]. Finally, Iacovelli et al. reported in third-line setting a median PFS and OS of 6.1 and 44.7 months, respectively [13]. Interestingly, when patients were stratified according to MSKCC prognostic criteria, the median OS was 59.9, 38.8, and 24.6 months in the good, intermediate, and poor prognosis groups, respectively, regardless of the treatment sequence. In this study, as a matter of fact, the VEGFi-VEGFi-mTORi sequence compared with the VEGFi-mTORi-VEGFi sequence was related to a better median total PFS (36.5 versus 29.3 months, *P* = 0.059) and OS (50.7 versus 37.8 months, *P* = 0.004). However, other retrospective studies have showed equal efficacy of these two sequences of treatments, with median OS generally not exceeding 30 months [14–16].

According to an algorithm treatment decision of changing the class of agent, having the patient received previous VEGFi-mTORi therapy, he should have been treated with another VEGFi, such as sorafenib.

Sorafenib is VEGFi-TKI approved to treat mRCC patients who have failed or cannot bear other therapies. Its main toxicity typically is hand-foot-skin (HFS) reaction that may be severe (grade 3-4) in about 15% of patients [17]. Particularly affected are hand, palm, and sole of the foot, leading in some cases to immobility.

Our patient was a musician; thus, considering the high risk of HFS occurrence, another target molecule was offered. Effectively, applying an* inverse decision-making* algorithm, as suggested by GOAL authors, we selected the best therapy by limiting toxicity and improving at the same time the treatment compliance [18].

Compared to other target therapies, such as everolimus, or axitinib, pazopanib showed clinical benefit as salvage therapy only in few studies [19, 20].

The first one included 28 patients treated with pazopanib after two previous therapies: median PFS was 16.5 months with grade 3-4 hypertension and proteinuria as the most common toxicities [19]. In the second one, pazopanib showed activity and safety also in patients with BM with up to 60% of SD and 13% of BM regression [20].

Our experience, coupled with data of literature, raises the question whether patients' clinical outcome was completely influenced by the presence of BM or rather by their exclusion from the most effective treatments such as the multimodality approach. Thus, in the evolving scenario of mRCC therapy, with no general consensus on the optimal sequence of therapies, the use of pazopanib in VEGFi-mTORi pretreated patients could represent a promising option.
